# Regulatory T Cell Suppressive Activity Predicts Disease Relapse During Disease-Modifying Anti-rheumatic Drug Dose Reduction in Rheumatoid Arthritis: A Prospective Cohort Study

**DOI:** 10.3389/fmed.2020.00025

**Published:** 2020-02-04

**Authors:** Korawit Kanjana, Parawee Chevaisrakul, Ponpan Matangkasombut, Karan Paisooksantivatana, Putthapoom Lumjiaktase

**Affiliations:** ^1^Department of Pathology, Faculty of Medicine, Ramathibodi Hospital, Mahidol University, Bangkok, Thailand; ^2^Division of Allergy, Immunology and Rheumatology, Department of Medicine, Faculty of Medicine, Ramathibodi Hospital, Mahidol University, Bangkok, Thailand; ^3^Department of Microbiology, Faculty of Science, Mahidol University, Bangkok, Thailand

**Keywords:** DMARD reduction, Foxp3^+^Treg, regulatory T cell, biomarkers, prognostic marker, rheumatoid arthritis

## Abstract

When the dose of conventional disease-modifying anti-rheumatic drugs (cDMARDs) is tapered in rheumatoid arthritis (RA) patients who achieve sustained remission, biomarkers for predicting disease relapse may be needed. A prospective, unblinded cohort study was conducted in nine RA patients with remission. Peripheral blood samples were collected at baseline and at 6, 12, and 24 weeks after cDMARD dose reduction (dose of combination regimens reduced to 50%) to determine the number of regulatory Foxp3^+^T cells (Tregs) and other T cell subpopulations as well as Treg suppressive activity. Additionally, plasma levels of 14 cytokines at each time-point were measured via flow cytometry. Univariate and multivariate analyses were performed to identify the factor(s) associated with RA relapse during the observational period. In univariate analysis, Treg suppression and DAS28 and VAS scores were associated with RA relapse after cDMARD dose tapering. However, in multivariate analysis, only Treg suppressive activity (<42%) was found to be an independent factor associated with RA relapse after cDMARD dose reduction to 50%. Of all patients who had ≥42% Treg suppressive activity during cDMAD reduction, three-fourth patients remained in the remission stage for 24 weeks. Treg suppressive activity (<42%) in RA patients with remission could be a potential biomarker for predicting RA relapse after cDMARD dose reduction, especially over a short-term period (24 weeks).

## Introduction

Rheumatoid arthritis (RA) is a chronic inflammatory form of arthritis that can progress to joint destruction and can involve organs, such as the eyes and lungs, as well as blood vessels. Disease-modifying anti-rheumatic drugs (DMARDs) are immunomodulating agents that can reduce joint inflammation and delay joint damage as part of a treatment plan targeting disease remission and/or symptom control in patients with low disease activity. RA patients require long-term treatment with DMARDs to maintain disease remission. Therefore, to avoid adverse effects of treatments in such cases, DMARD dose reduction has been proposed for RA patients with sustained remission (1). Many predictors, such as multi-biomarker disease activities, serological tests and ultrasound or magnetic resonance imaging have been proposed as prognostic tools for detecting subclinical synovitis, with limited success (2–4).

Because RA is an autoimmune disease, immunological biomarkers may be promising indicators for predicting disease relapse during DMARD dose tapering. Foxp3^+^ regulatory T cells (Tregs), a subpopulation of CD4^+^ T lymphocytes, are mainly involved in immunosuppression. Alterations in Foxp3^+^ Treg numbers have been reported in different stages of RA (5), and Treg numbers have been found to increase in RA patients with remission (6). As cellular plasticity of Treg/Th17 can occur at sites of inflammation, the suppressive function of Tregs, rather than Treg numbers, may indicate the status of immune tolerance in RA patients (7, 8). This has been confirmed in a previous study comparing Treg suppressive activity between healthy patients and patients with different stages of RA (9).

This study was conducted to evaluate biomarkers that may help predict RA relapse after conventional DMARD (cDMARD) 50%-dose reduction, such as Treg numbers and functions, as well as levels of several cytokines in RA patients with sustained remission.

## Materials and Methods

### Participants and Study Design

Nine patients diagnosed with RA according to the ACR/EULAR 2010 or ACR 1987 classification criteria (10) were enrolled in this prospective, unblinded cohort study. The study was conducted at the Ramathibodi Hospital, Mahidol University, Bangkok, Thailand, and was approved by the Ethics Committees on Human Rights Related to Research Involving Human Subjects of Faculty of Medicine, Ramathibodi Hospital, in accordance with the Declaration of Helsinki (ID: 10-57-37). Inclusion criteria were RA patients without clinical synovitis and with disease activity evaluated using DAS28 score of <2.6 (DAS28 remission) as well as sustained clinical remission for at least 6 months before enrolment. Exclusion criteria were RA patients with subclinical synovitis defined by Doppler ultrasound during 6 months prior to the start of the study, biological DMARD or targeted therapy use, corticosteroid use, active infection at 2 weeks prior to the study and a history of cancer or immune deficiency. A DMARD reduction regimen was adopted from the RETRO study (11) in which 50% dose reduction of each cDMARD was applied for patients taking combination cDMARDs. Patients were followed up for 24 weeks or until disease relapse (defined by arthritis symptoms and a DAS28 > 2.6). Blood samples were collected at baseline, prior to cDMARD reduction and at 6, 12, and 24 weeks or until disease relapse. In RA patients with disease relapse, cDMARDs were restarted at the previous dose before tapering. A detailed description of cDAMRD reduction is given in Table S1.

### Cell Sorting and Co-culture Suppression Assay

Plasma was prepared from the patients' fresh whole blood (16 mL) and stored at −80°C until used for cytokine panel detection via flow cytometry (LEGENDplex; BioLegend, USA). Peripheral blood mononuclear cells (PBMCs) were isolated using a commercial isolation kit (CPT tube; Becton Dickinson, USA), followed by CD8^+^ cell depletion using a magnetic column method (X-Zell Biotec, TH). CD4^+^ cells were stained with conjugated anti-CD4PEcy7, -CD25PEcy5, and -CD127PE murine antibodies (Becton Dickinson, USA) for cell sorting of Treg (CD4^+^CD25^high+^CD127^low−^) and conventional T cell or Tconv (CD4^+^CD25^−^CD127^+^) sorting using FACSAria III (Becton Dickinson, USA). Tregs were subjected to short-term expansion for 4 days at 37°C under a humidified atmosphere containing 5% CO_2._ Briefly, 1 × 10^6^ Tregs/well were stimulated by CD3/CD28 bead (cat 111.31D, Life Technologies, USA). Culture medium (RPMI-1640 with glutamine, Life Technologies, USA) for the assay is supplemented with 10% fetal bovine serum, 1% penicillin/streptomycin, 1% HEPES buffer, 1% Sodium pyruvate, 0.1% 2-mercaptoethanol (Life Technologies, USA), and 500 IU/mL recombinant human interleukin 2 (rIL-2) (ImmunoTools, Germany). The final volume for expansion is 200 μl/well. The subsequent phenotype was confirmed through intracellular Foxp3 staining. Autologous Tconv were maintained in parallel in a culture medium supplemented with 500 IU/mL rIL-2 for 4 days as described above. “Rested” Tconv were labeled with carboxyfluorescein succinimidyl ester (CFSE) (Life Technologies, USA) prior to co-culturing with “expanded” Treg at Treg:Tconv ratios of 10:1 and 5:1. The details of this method validation is given in our previous study (12). The ratio of 10:1 was used for monitoring Treg suppressive activity in this study. Furthermore, 500 IU/mL rIL-2 (ImmunoTools, Germany) and anti-CD3/CD28 beads (Life Technologies, USA) (at 1:2 bead:cell ratio) were added and co-cultivation was performed at 37°C under 5% CO_2_ atmosphere for 3 days. The proliferation of CFSE-labeled Tconv was determined via flow cytometry (Beckman Coulter), and Treg suppressive activity was calculated as the percentage of suppression using the following formula: %suppression = 100 – ([% Tconv proliferation in co-culture]/[%Tconv proliferation in the absence of Treg]) × 100.

### Foxp3^+^Treg and T Cell Subset Identification

PBMCs without CD8^+^ depletion were used to study the number of Foxp3^+^ Tregs and T cell subsets via flow cytometry. Intracellular Foxp3 were stained with rat anti-human conjugated Foxp3eflu660 antibodies (Affymetrix ebioscience, USA) and the CD4^+^CD25^+^Foxp3^+^ population was defined as Foxp3^+^ Treg, whereas CD4^+^CD25^+^, CD4^+^CD25^+^CD127^+^ and CD4^+^CD25^high+^CD127^low−^ populations were defined as T cell subpopulation types. The numbers of Treg and T cells were determined via flow cytometry. The gating strategies used have been reported in our previous study (12).

### Statistical Analysis

Descriptive results are presented as the mean, median, standard deviation (SD) and interquartile range (IQR). Treg suppressive activity, numbers of Tregs and T cell subsets and plasma cytokine levels were compared between RA patients with relapse and those with sustained remission at 24 weeks using *t*-test and Mann–Whitney *U*-test or Wilcoxon signed-rank test. Time to relapse was determined using Kaplan–Meier analysis. The cut-off value of Treg suppressive activity (%) between RA patients with relapse and those with sustained remission was calculated using Cox regression analysis. Immunological markers associated with relapse were analyzed through univariate logistic regression analysis using the results of sustained remission and relapse status. Parameters with *p*-value < 0.05 in univariate analysis were included in the backward selection for multivariate logistic regression analysis. *P-*value < 0.05 was considered to be significant. All statistical analyses were performed using IBM SPSS version 16.00 for Windows OS (SPSS Inc., USA).

## Results

### Patients' Characteristics

All nine RA patients had established RA with a median disease duration of 7 years (range, 4–11 years). Eight patients were treated with cDMARDs in the first year of appearance of symptoms. The clinical course of the nine patients regarding symptom onset, disease duration, bone erosion and extra-articular manifestation is described in the Supplementary Data. There were no significant differences in sex, average age, baseline DAS28 score, and others laboratory parameter between RA patients with sustained remission and relapse (Table 1).

**Table 1:** Baseline characteristics of rheumatoid arthritis patients enrolled in the study.

**Variables**	**Sustained remission during DMARD dose reduction**	**Relapse during DMARD dose reduction**	***P*-values**
Percentage of all patients	33.3% (*n* = 3)	66.7% (*n* = 6)	–
Age (years), mean ± SD	56.0 ± 14.9	53.8 ± 11.9	1.000
WBC count (cells/mm^3^), mean ± SD	6.42 ± 1.49	5.57 ± 1.79	0.439
Lymphocyte count (cells/mm^3^), mean ± SD	29.67 ± 10.97	26.50 ± 6.09	0.584
ESR (mm/h), mean ± SD	30.00 ± 10.00	19.17 ± 12.15	0.227
Serology positive (%) (ACPA and/or RF)	33.3% (1/3)	66.7% (4/6)	0.317
VAS, median ± IQR	0.00	0.00 ± 12.50	0.289
DAS28, mean ± SD	2.35 ± 0.24	1.96 ± 0.68	0.397
Baseline DMARDs used (%)			
2-DMARDs combined	–	50%	–
3-DMARDs combined	100%	50%	0.317

*ACPA, anti-citrullinated protein antibody; DAS28, disease activity score-28; DMARD, disease-modifying anti-rheumatic drug; ESR, erythrocyte sedimentation rate; IQR, interquartile range; RF, rheumatoid factor; VAS, visual analog scale; WBC, white blood cell*.

### Foxp3^+^Tregs, T Cell Subpopulations, and Foxp3^+^Treg Ratios

**Figure 1:**
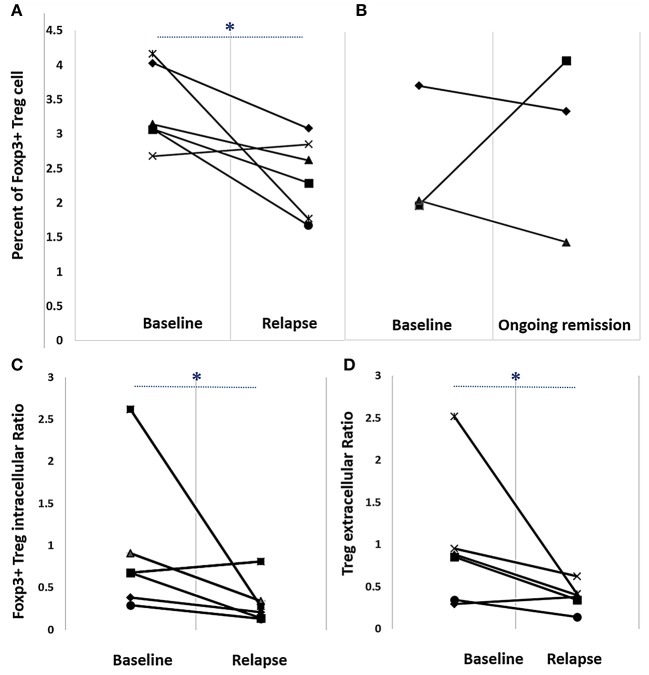
Percent Foxp3^+^Treg numbers **(A,B)** and Foxp3^+^Treg intracellular **(C)** and extracellular **(D)** ratios in rheumatoid arthritis patients on a reduced disease-modifying anti-rheumatic drug regimen. Cells were labeled with appropriate fluorescent-labeled antibodies and analyzed via flow cytometry. **(A)** Baseline and relapse percent Foxp3^+^Treg numbers [median ± interquartile range (IQR)] were 3.11 ± 1.1 and 2.5 ± 1.1, respectively. **(B)** Baseline and ongoing remission percent Foxp3^+^Treg (median ± IQR) numbers were 2.04 ± 0.9 and 3.35 ± 1.1, respectively. **(C)** Baseline and relapse Foxp3^+^Treg intracellular ratio (Foxp3^+^Treg:CD4^+^ CD25^+^CD127^+^) (median ± IQR) was 0.7 ± 1.0 and 0.2 ± 0.3, respectively. **(D)** Baseline and relapse Foxp3^+^Treg extracellular ratio (CD4 CD25^high+^ CD127^low−^:CD4 CD25^+^ CD127^+^) (median ± IQR) was 0.9 ± 1.0 and 0.4 ± 0.2, respectively. *p < 0.05, Wilcoxon signed-rank test.

T cell subpopulation numbers were reported as a percentage of total CD4^+^ T cells. Foxp3^+^Treg numbers at baseline before cDMARD reduction were significantly higher than those observed at the relapse visit (Figure 1A); however, no difference was observed in patients with sustained remission at 24 weeks (Figure 1B). There was no difference in T cell subpopulation numbers CD4^+^CD25^+^, CD4^+^CD25^+^CD127^+^ and CD4^+^CD25^high+^CD127^low−^ between patients with sustained remission and those with relapse during the observational period (data not shown). In RA patients with relapse, effector/activated T cell (CD4^+^CD25^+^ and CD4^+^CD25^+^CD127^+^) subpopulation numbers were slightly increased at the relapse visit compared with those at baseline (from 10.02 ± 4.51 to 13.17 ± 7.18% and from 4.23 ± 7.73 to 8.65 ± 7.80%, respectively), but these numbers remained unchanged in patients with sustained remission. Both Foxp3^+^Treg intracellular (Foxp3^+^Treg:CD4^+^CD25^+^CD127^+^) and Foxp3^+^Treg extracellular (CD4^+^CD25^high+^CD127^low−^:CD4^+^CD25^+^CD127^+^) ratios were significantly decreased between baseline and relapse stage (0.68 ± 0.98 to 0.25 ± 0.32% and 0.87 ± 1.02 to 0.39 ± 0.17%, respectively) (Figures 1C,D).

### Treg Suppressive Activity

**Figure 2:**
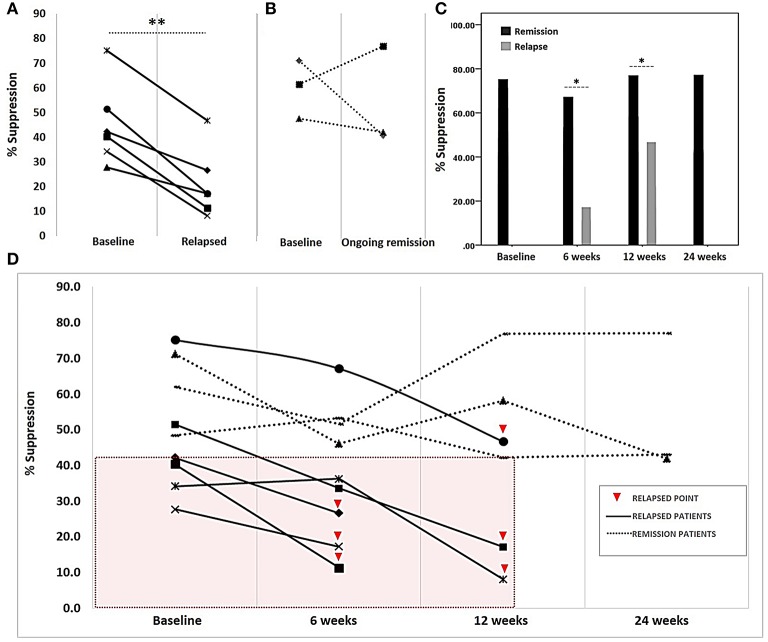
Treg suppressive activity in rheumatoid arthritis patients on a reduced disease-modifying anti-rheumatic drug regimen. Following co-cultivation of Tregs and autologous Tconv for 3 days, the proliferation of carboxyfluorescein succinimidyl ester (CFSE)-labeled Tconv was determined via flow cytometry. Treg suppressive activity, presented as percent suppression (100 – [%Tconv proliferation in co-culture]/[%Tconv proliferation in the absence of Treg] × 100), was determined at the time of drug regimen initiation (baseline) and at 6, 12, and 24 weeks thereafter. **(A)** Comparison of suppressive activity at baseline and disease relapse. **(B)** Suppressive activity in patients with ongoing remission at baseline and at the final visit at 24 weeks after drug reduction. **(C)** Comparison of suppressive activity between patients with ongoing remission and relapse during follow-up visits (6 and 12 weeks). **(D)** Suppressive activity in each patient (*n* = 9) at different time-points; boxed area (pink) indicates suppressive activities in only those patients with relapse who had a suppressive activity of <42%. Treg suppressive activity in all patients at the initiation of the study (baseline), patients in relapse at week 6 post-initiation (6 weeks), patients in relapse at week 12 post-initiation (12 weeks) and patients with ongoing remission at week 24 post-initiation (24 weeks). A comparison of suppressive activity at baseline and that after relapse was performed using a paired sample *t-*test. The comparison of suppressive activity at four visits and different visits was analyzed using Kruskall–Wallis and Mann–Whitney *U*-tests, respectively. Significance at *p*-value: *p < 0.05 and **p < 0.01.

After Treg expansion, Treg and rested autologous Tconv were co-cultured at a ratio of 10:1 for 3 days. We observed a 2-fold decrease in Treg suppressive activity compared with that baseline in patients with relapse after DMARD dose reduction (45.21 ± 16.72 to 21.19 ± 14.01%; 95% CI: 14.51–33.53; *p* = 0.001) (Figure 2A). Conversely, Treg suppressive activity in patients with sustained remission remained stable when compared with that at baseline (Figure 2B). When Treg suppressive activity was compared between patients with relapse and sustained remission at baseline and at 6 and 12 weeks, it was found that Treg suppression was 50.32 ± 16.33% at baseline. In three patients with sustained remission over the course of 24 weeks, Treg suppressive activity remained at a high level (54.04 ± 20.03%), whereas this activity in patients with relapse at 6 or 12 weeks was lower than that at baseline (Figure 2D). In RA patients with relapse, Treg suppressive activity declined, particularly in comparison with the activity in those with sustained remission at each time-point of relapse (14.24 ± 4.21 vs. 48.01 ± 12.33%; *p* = 0.011 at 6 weeks and 24.66 ± 16.53 vs. 59.14 ± 17.32% at 12 weeks; *p* < 0.05) (Figure 2C). Treg suppressive activity and cDMARD dose reduction regimen in each patient is shown in Table S1.

### Plasma Cytokine Levels

The levels of 14 plasma cytokines were measured via flow cytometry, including both pro-inflammatory and anti-inflammatory cytokines (IFN-γ, IL-2, IL-4, IL-5, IL-6, IL-9, IL-10, IL-13, IL-17A, IL-17F, IL-21, IL-22, TGF-β, and TNF-α). In RA patients with relapse, it was observed that IFN-γ, IL-10, IL-21, and TNF-α levels at disease relapse visits were significantly higher than those at baseline, before cDMARD dose tapering (Table 2). In RA patients with sustained remission, there was no change in the levels of all cytokines measured from baseline to 24 weeks (Table S2). However, IL-17A levels measured at relapse visits at 12 weeks were higher than those at baseline (*p* < 0.05). All significantly altered cytokine levels are summarized in the line graph in Supplementary Figure 1. Therefore, univariate logistic regression analysis did not reveal any cytokine as a predictive factor for disease relapse in this study (Table S3).

**Table 2:** Cytokine levels in rheumatoid arthritis patients with relapse (*n* = 6).

**Cytokines (pg/mL), median ± IQR**	**Baseline**	**Relapsed**	***P*-values**
IL-2	2 ± 2	2 ± 12	0.180
IL-4	20 ± 21	38 ± 170	0.075
IL-6	4 ± 4.	8 ± 22	0.075
IL-9	2 ± 1	3 ± 9	0.075
IL-10	36 ± 23	71 ± 71.4	0.028^*^
IL-13	<2	3 ± 14	0.109
IL-17A	<2	6 ± 30	0.068
IL-17F	2 ± 6	3 ± 6	0.080
IL-21	5 ± 10	12 ± 43	0.028^*^
IL-22	28 ± 15	34 ± 22	0.600
IFN-γ	8 ± 12	15 ± 22	0.028^*^
TNF-α	16 ± 29	21 ± 84	0.028^*^
TGF-β (ng/mL), mean ± SD	326 ± 355	348 ± 389	0.305

* *Significance at p < 0.05. IFN-γ, interferon-γ; IL, interleukin; IQR, interquartile range; TGF-β, transforming growth factor β; TNF-α, tumor necrosis factor α. IL-5 level was <1.95 pg/mL at all visits (data not shown)*.

### Kaplan–Meier Plot and Univariate Logistic Regression Analysis

**Figure 3:**
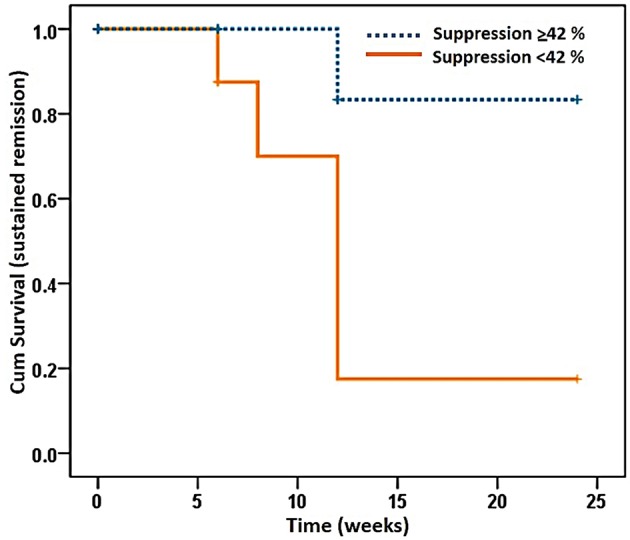
Kaplan–Meier plot of a proportion of rheumatoid arthritis patients in ongoing remission over the study period of 24 weeks. Patients were divided into those with a Treg suppressive activity (Suppression) of <42 and ≥42%. Percentage of Treg suppressive activity was determined as described in the legend to Figure 2. *P* < 0.05; 95% confidence interval: 9.64–14.36, Kaplan–Meier analysis.

Cox regression analysis showed that the cut-off value of Treg suppressive activity that could predict relapse of disease in RA patients in this study was 42%. A survival analysis using a Kaplan–Meier plot demonstrated that 80% of RA patients who had Treg suppressive activity > 42% at baseline had sustained disease remission for 24 weeks following cDMARD dose reduction (95% CI 9.64–14.36; *p* < 0.05) (Figure 3). Univariate analysis showed that three parameters including Treg suppressive activity (>42%), DAS28 score and VAS score (Table S3) could predict RA relapse after DMARD dose tapering. However, multivariate analysis (by logistic regression) (Table S4) showed only Treg suppressive activity (>42%) to be the independent parameter that could predict RA relapse after cDMARD dose reduction (odds ratio: 0.843, 95% CI 0.726–0.978; *p* < 0.05).

## Discussion

The study results revealed that Treg suppressive activity may be a predictive factor of disease relapse in RA patients with sustained remission. However, the cDMARD dose tapering protocol used in the study allowed only short-term observation. More than one-third of patients may experience disease relapse after a 50% dose reduction of cDMARDs, particularly in the first 6 months (11). Increased Treg numbers were reported in RA patients after 4 weeks of treatment with a combination of cDMARDs and an anti-TNF from among etanercept, infliximab, or adalimumab (6). Impaired Treg suppressive activity in patients with active RA may be improved by MTX treatment through the demethylation of *FOXP3*, leading to an increased expression of Foxp3 and cytotoxic T-lymphocyte–associated antigen 4 (13). Nonetheless, in the small number of patients taking different combinations of cDMARDs, we were not able to address the impact of cDMARD dose tapering on the likelihood of RA relapse.

Several studies have supported the notion that Tregs influence RA in different stages of the disease. For example, Kawashiri et al. demonstrated lower numbers of peripheral blood CD4^+^CD25^high+^CD127^low−^ Treg populations in RA patients with active disease than in RA patients in remission (14). In addition, Treg suppressive activity in the synovial fluid has been shown to be impaired in patients with active RA, which could be influenced by the hyperactivation of protein kinase B, resulting in T cells becoming resistant to Treg suppression (15). One study found that the Treg/Th17 ratio increased after the second month of treatment with an IL-6 receptor inhibitor in RA patients (16). However, Treg numbers alone may not be sufficient to reflect the immunosuppressive status due to Treg/Th17 cellular plasticity in RA. For this reason, measuring Treg suppressive activity might be useful in clinical practice (17).

In this study, we demonstrated that Treg suppressive activity in RA patients with relapse significantly decreased at relapse visits compared with that at baseline after cDMARD dose reduction. Interestingly, we found that Treg suppressive activity <42% at baseline could predict disease relapse over a 24 weeks observation period. In contrast, Treg suppressive activity (>42%) was stable during the 24 weeks of this study in RA patients with sustained remission after drug tapering. This may reflect that while drugs acted *in vivo* as immunosuppressants, immune tolerance in some patients also influenced the effect of cDMARDs.

Pro-inflammatory cytokines from autologous effector T cells inhibit Treg suppressive activity, and this activity was recovered after anti-TNF therapy (18). In active stages of RA, these cytokines facilitate T-cell-mediated cellular immunity, monocyte activation, Treg-plasticity and auto-antibody production, leading to immune deregulation (19). For example, TNF-α binds to TNF receptor type II on Tregs, resulting in the downregulation of FOXP3 expression by inducing protein phosphatase 1 (PP1) expression (20). IL-10 is a suppressive cytokine produced by Tregs, and increased IL-10 mRNA expression has been previously shown in RA patients (21) Increased serum IL-10 levels have also been found in RA patients compared with those in healthy controls. However, IL-10 is also produced by synovial mononuclear cells (MNCs), such as monocytes, macrophages and Th1 cells, as well as some pro-inflammatory cytokines, including TNFα, IL-1, and IL-6 (22). This may be the reason why IL-10 levels were increased, whereas Treg numbers and activity were impaired in patients with active RA. We also observed that levels of many inflammatory cytokines, such as IFN-γ, IL-10, IL-17A, IL-21, and TNF-α were elevated in RA patients with relapse. However, we were not able to demonstrate an association between these cytokines and disease relapse.

Only one-third of the RA patients in the study remained in the remission stage for 24 weeks after a 50% dose reduction of cDMARDs. Treg suppressive activity <42% may be a predictor of disease relapse. The limitations of this study were its small sample size and limited study duration. A multivariate analysis may not be appropriate for interpreting the results of this study. We found that Treg suppressive activity can be potentially used as a predictive tool for RA relapse when considering DMARD dose tapering. This study lays the foundation for further investigations involving a larger cohort over a longer period in RA patients.

## Data Availability Statement

The raw data supporting the conclusions of this article will be made available by the authors, without undue reservation, to any qualified researcher.

## Ethics Statement

The studies involving human participants were reviewed and approved by Ethics Committees of Faculty of Medicine, Ramathibodi Hospital, Mahidol University (ID: 10-57-37). The patients/participants provided their written informed consent to participate in this study.

## Author Contributions

KK and PC contributed to data collection and patient enrolment. PL contributed as funding provider and to overall supervision. All authors contributed to the design and interpretation of the study and also prepared the manuscript.

### Conflict of Interest

The authors declare that the research was conducted in the absence of any commercial or financial relationships that could be construed as a potential conflict of interest.

## References

[1] Schett G Emery P Tanaka Y Burmester G Pisetsky DS Naredo E. Tapering biologic and conventional DMARD therapy in rheumatoid arthritis: current evidence and future directions. Ann Rheum Dis. (2016) 75:1428. 10.1136/annrheumdis-2016-209201 27261493

[2] Hirata S Dirven L Shen Y Centola M Cavet G Lems WF. A multi-biomarker score measures rheumatoid arthritis disease activity in the BeSt study. Rheumatology. (2013) 52:1202–7. 10.1093/rheumatology/kes362 23392591 PMC3685330

[3] Hafström I Engvall IL Rönnelid J Boonen A van der Heijde D Svensson B Rheumatoid factor and anti-CCP do not predict progressive joint damage in patients with early rheumatoid arthritis treated with prednisolone: a randomised study. BMJ Open. (2014) 4:e005246 10.1136/bmjopen-2014-005246 PMC4120364 25079933

[4] Kawashiri S-Y Fujikawa K Nishino A Okada A Aramaki T Shimizu T. Ultrasound-detected bone erosion is a relapse risk factor after discontinuation of biologic disease-modifying antirheumatic drugs in patients with rheumatoid arthritis whose ultrasound power doppler synovitis activity and clinical disease activity are well controlled. Arthritis Res Therapy. (2017) 19:108. 10.1186/s13075-017-1320-2 28545509 PMC5445491

[5] Morita T Shima Y Wing JB Sakaguchi S Ogata A Kumanogoh A. The proportion of regulatory T cells in patients with rheumatoid arthritis: a meta-analysis. PLoS ONE. (2016) 11:e0162306. 10.1371/journal.pone.0162306 27622457 PMC5021283

[6] Szalay B Vásárhelyi B Cseh Á Tulassay T Deák M Kovács L. The impact of conventional DMARD and biological therapies on CD4^+^ cell subsets in rheumatoid arthritis: a follow-up study. Clin Rheumatol. (2014) 33:175–85. 10.1007/s10067-013-2352-x 23934385

[7] Hoechst B Gamrekelashvili J Manns MP Greten TF Korangy F. Plasticity of human Th17 cells and iTregs is orchestrated by different subsets of myeloid cells. Blood. (2011) 117:6532. 10.1182/blood-2010-11-317321 21493801

[8] Kleinewietfeld M Hafler DA. The plasticity of human Treg and Th17 cells and its role in autoimmunity. Semin Immunol. (2013) 25:305–12. 10.1016/j.smim.2013.10.009 24211039 PMC3905679

[9] Kanjana K Chevaisrakul P Matangkasombut P Paisooksantivatana K Lumjiaktase P SAT0007 the suppressive activity of peripheral blood treg represents immunological remission in rheumatoid arthritis patients. Ann Rheumat Dis. (2019) 78(Suppl. 2):1068 10.1136/annrheumdis-2019-eular.1487

[10] Kay J Upchurch KS. ACR/EULAR 2010 rheumatoid arthritis classification criteria. Rheumatology. (2012) 51(suppl. 6):vi5–9. 10.1093/rheumatology/kes279 23221588

[11] Haschka J Englbrecht M Hueber AJ Manger B Kleyer A Reiser M. Relapse rates in patients with rheumatoid arthritis in stable remission tapering or stopping antirheumatic therapy: interim results from the prospective randomised controlled RETRO study. Ann Rheumat Dis. (2016) 75:45. 10.1136/annrheumdis-2014-206439 25660991

[12] Kanjana K Paisooksantivatana K Matangkasombut P Chevaisrakul P Lumjiaktase P. Efficient short-term expansion of human peripheral blood regulatory T cells for co-culture suppression assay. J Immunoass Immunochem. (2019) 40:573–89. 10.1080/15321819.2019.1659813 31460830

[13] Cribbs AP Kennedy A Penn H Amjadi P Green P Read JE. Methotrexate restores regulatory T cell function through demethylation of the FoxP3 upstream enhancer in patients with rheumatoid arthritis. Arthritis Rheumatol. (2015) 67:1182–92. 10.1002/art.39031 25604080

[14] Kawashiri S-Y Kawakami A Okada A Koga T Tamai M Yamasaki S CD4^+^CD25^high^CD127^low/−^ Treg cell frequency from peripheral blood correlates with disease activity in patients with rheumatoid arthritis. J Rheumatol. (2011) 38:2517 10.3899/jrheum.110283 21921095

[15] Cooles FAH Isaacs JD Anderson AE. Treg cells in rheumatoid arthritis: an update. Curr Rheumatol Rep. (2013) 15:352. 10.1007/s11926-013-0352-0 23888361

[16] Pesce B Soto L Sabugo F Wurmann P Cuchacovich M López MN. Effect of interleukin-6 receptor blockade on the balance between regulatory T cells and T helper type 17 cells in rheumatoid arthritis patients. Clin Exp Immunol. (2013) 171:237–42. 10.1111/cei.12017 23379428 PMC3569529

[17] Wang T Sun X Zhao J Zhang J Zhu H Li C. Regulatory T cells in rheumatoid arthritis showed increased plasticity toward Th17 but retained suppressive function in peripheral blood. Ann Rheumat Dis. (2015) 74:1293. 10.1136/annrheumdis-2013-204228 24521740

[18] Ehrenstein MR Evans JG Singh A Moore S Warnes G Isenberg DA. Compromised function of regulatory T cells in rheumatoid arthritis and reversal by anti-TNFalpha therapy. J Exp Med. (2004) 200:277–85. 10.1084/jem.20040165 15280421 PMC2211983

[19] Brzustewicz E Bryl E. The role of cytokines in the pathogenesis of rheumatoid arthritis–practical and potential application of cytokines as biomarkers and targets of personalized therapy. Cytokine. (2015) 76:527–36. 10.1016/j.cyto.2015.08.260 26321413

[20] Nie H Zheng Y Li R Guo TB He D Fang L. Phosphorylation of FOXP3 controls regulatory T cell function and is inhibited by TNF-α in rheumatoid arthritis. Nat Med. (2013) 19:322. 10.1038/nm.3085 23396208

[21] Alanärä T Karstila K Moilanen T Silvennoinen O Isomäki P. Expression of IL-10 family cytokines in rheumatoid arthritis: elevated levels of IL-19 in the joints. Scand J Rheumatol. (2010) 39:118–26. 10.3109/03009740903170823 20001767

[22] St Clair EW. Interleukin 10 treatment for rheumatoid arthritis. Ann Rheumat Dis. (1999) 58(suppl. 1):I99. 10.1136/ard.58.2008.i99 10577984 PMC1766579

