# Supplementary material for: Regulatory T Cell Suppressive Activity Predicts Disease Relapse During Disease-Modifying Anti-rheumatic Drug Dose Reduction in Rheumatoid Arthritis: A Prospective Cohort Study
Source: Front Med (Lausanne). 2020 Feb 4;7:25. doi: 10.3389/fmed.2020.00025 (PMC7011614; doi:10.3389/fmed.2020.00025)
Supplement: Supplementary file 1 [file Data_Sheet_1.docx]

Supplementary Material

# Table S1. Treg suppressive activity and reduced disease-modifying anti-rheumatic drug (DMARD) regimen of rheumatoid arthritis patients

| Patient ID* | Suppressive activity (%) | DAS  28 | VAS | ESR | Serology | Reduced DMARD regimen | | | | |
| --- | --- | --- | --- | --- | --- | --- | --- | --- | --- | --- |
|  |  |  |  |  |  | MTX, mg/wk | LEF, mg/wk | SSZ, mg/day | HCQ, mg/wk | AZA, mg/wk |
| DT001-1^st^ | 42 | 2.3 | 0.0 | 27 | Negative | 10 | 80 | - | - | - |
| DT001-3^rda^ | 26 | 2.8 | 0.0 | 57 | - | Re-initiation original DMARD regimen | | | | |
| DT002-1^st^ | 40 | 1.9 | 0.0 | 15 | Positive | 20 | 40 | 2,000 | - | - |
| DT002-2^nda^ | 11 | 3.4 | 50.0 | 14 | - |  | | | | |
| DT003-1^st^ | 71 | 2.6 | 0.0 | 40 | Negative | 10 | - | 500 | 1,000 | - |
| DT003-2^nd^ | 46 | 2.6 | 0.0 | 43 | - | 5 | - | 250 | 500 | - |
| DT003-3^rd^ | 58 | 2.4 | 0.0 | 33 | - | 5 | - | 250 | 500 | - |
| DT003-4^thb^ | 42 | 2.6 | 0.0 | 41 | - | 5 | - | 250 | 500 | - |
| DT004-1^st^ | 28 | 2.7 | 20.0 | 32 | Positive | 20 | 80 | - | 250 | - |
| DT004-2^nda^ | 17 | 3.5 | 30.0 | 24 | - | Re-initiation original DMARD regimen | | | | |
| DT005-1^st^ | 34 | 1.0 | 0.0 | 4 | Negative | 15 | 80 | - | - | - |
| DT005-2^nd^ | 36 | 0.8 | 0.0 | 3 | - | 7.5 | 40 | - | - | - |
| DT005-3^rda^ | 8 | 3.0 | 50.0 | 25 | - | Re-initiation original DMARD regimen | | | | |
| DT006-1^st^ | 75 | 2.5 | 10.0 | 30 | Positive | 15 | - | - | 1,000 | - |
| DT006-2^nd^ | 67 | 2.2 | 10.0 | 20 | - | 7.5 | - | - | 500 | - |
| DT006-3^rda^ | 47 | 3.2 | 30.0 | 34 | - | Re-initiation original DMARD regimen | | | | |
| DT007-1^st^ | 51 | 1.4 | 0.0 | 7 | Positive | 5 | - | 500 | - | 100 |
| DT007-2^nd^ | 33 | 1.7 | 10.0 | 10 | - | 2.5 | - | 250 | - | 50 |
| DT007-3^rda^ | 17 | 2.3 | 50.0 | 10 | - | Re-initiation original DMARD regimen | | | | |
| DT008-1^st^ | 62 | 2.4 | 0.0 | 30 | Positive | 10 | 80 | - | 1,500 | - |
| DT008-2^nd^ | 52 | 1.7 | 0.0 | 12 | - | 5 | 40 | - | 750 | - |
| DT008-3^rd^ | 77 | 1.6 | 0.0 | 10 | - | 5 | 40 | - | 750 | - |
| DT008-4^thb^ | 77 | 2.6 | 20.0 | 14 | - | 7.5 | 60 | - | 750 | - |
| DT009-1^st^ | 48 | 2.1 | 0.0 | 20 | Negative | 15 | 160 | 2,000 | - | - |
| DT009-2^nd^ | 53 | 2.0 | 0.0 | 17 | - | 7.5 | 80 | 1,000 | - | - |
| DT009-3^rd^ | 42 | 2.2 | 0.0 | 25.0 | - | 7.5 | 80 | 1,000 | - | -* |
| DT009-4^thb^ | 43 | 2.1 | 0.0 | 22.0 | - | 7.5 | 80 | 1,000 | - | - |

*n^th^ refers to the visit following reduced DMARD dose regimen

^a^Relapse after reduced DMARD dose regimen

^b^Ongoing remission on week 24 post-reduced DMARD dosage regimen

AZA, azathioprine; DAS28, disease activity score-28; ESR, erythrocyte sedimentation rate; HCQ, hydroxychloroquine; LEF, leflunomide; MTX, methotrexate; SSZ, sulfasalazine; VAS, visual analogue scale; Serology, anti-citrullinated protein antibodies and/or rheumatoid factor

Note: Suppressive activity of DT001^2nd^ visit was not assessed.

| Cytokines  (pg/mL), median ± IQR | Baseline | Remission | *p*-values |
| --- | --- | --- | --- |
| IL-4 | 20.21 ± 21.16 | 35.51 ± 177.78 | 0.309 |
| IL-6 | 4.01 ± 4.02 | 6.52 ± 3.94 | 0.301 |
| IL-9 | 2.08 ± 1.34 | 3.71 ± 1.01 | 0.051 |
| IL-10 | 35.90 ± 23.11 | 42.02 ± 3.94 | 0.782 |
| IL-17F | 2.42 ± 5.65 | 6.46 ± 3.50 | 0.301 |
| IL-21 | 5.46 ± 10.18 | 13.33 ± 8.61 | 0.405 |
| IL-22 | 28.27 ± 14.98 | 42.68 ± 103.77 | 0.167 |
| IFN-γ | 8.27 ± 12.13 | 14.44 ± 6.55 | 0.309 |
| TNF-α | 16.21±29.18 | 34.53 ± 9.02 | 0.165 |
| TGF-β, ng/mL (mean ± SD) | 321.04 ± 210.82 | 283.57 ± 248.54 | 0.803 |

# Table S2. Cytokine levels in rheumatoid arthritis patients with ongoing remission (n = 3)

*Significance at p-value<0.05. IFN-γ, interferon-γ; IL, interleukin; IQR, interquartile range; TGF-β, transforming growth factor β; TNF-α, tumour necrosis factor-α

Note: IL-2, IL-5, IL-13 and IL-17A levels were lower than the limit of detection at all visits (data not show)

# Table S3 Univariate analysis: potential variables associated with a flare during DMARDs reduction

| Parameters/Markers | OR | 95% CI | P-value |
| --- | --- | --- | --- |
| *T cell properties* | | |  |
| Foxp3+Tregs | 0.282 | 0.047–1.699 | 0.167 |
| CD4+CD25high+CD127low− | 0.359 | 0.050–2.156 | 0.263 |
| CD4+CD25+CD127+ | 1.175 | 0.684–2.017 | 0.560 |
| CD4+CD25+ | 1.166 | 0.708–1.922 | 0.546 |
| Foxp3+Treg intracellular ratio | 8.150 | 0.003–2.638 | 0.611 |
| Treg extracellular ratio | 0.007 | 0.000–1.185 | 0.058 |
| Treg suppressive activity | 0.841 | 0.731–0.968 | 0.016* |
| *Plasma cytokines* | | |  |
| IL-2 | 0.248 | 0.035–1.777 | 0.165 |
| IL-4 | 0.934 | 0.726–1.201 | 0.595 |
| IL-6 | 1.204 | 0.931–1.556 | 0.157 |
| IL-9 | 1.206 | 0.689–2.110 | 0.512 |
| IL-10 | 1.058 | 0.998–1.116 | 0.058 |
| IL-13 | 3.161 | 0.734–13.606 | 0.122 |
| IL-17A | 11.542 | 0.562–237.023 | 0.113 |
| IL-17F | 0.614 | 0.113–2.830 | 0.532 |
| IL-21 | 1.120 | 0.795–1.579 | 0.517 |
| IL-22 | 1.095 | 0.788–1.521 | 0.590 |
| IFN-Ƴ | 1.078 | 0.859–1.352 | 0.517 |
| TNF-α | 0.991 | 0.923–1.064 | 0.805 |
| TGF-β | 1.003 | 0.997–1.008 | 0.373 |
| *Others* | | |  |
| Age | 1.049 | 0.946–1.162 | 0.366 |
| WBC | 1.320 | 0.745–2.341 | 0.342 |
| Lymphocyte | 0.882 | 0.756–1.028 | 0.108 |
| ESR | 0.965 | 0.862–1.081 | 0.539 |
| ACPA/RF positive | 1.183 | 0.891–1.573 | 0.246 |
| DAS28 | 1.201 | 1.051–9.543 | 0.037* |
| VAS | 1.164 | 1.034–1.210 | 0.012* |

*Significance at *p*-value<0.05. Tregs: regulatory T cells, WBC: white blood cell count, ESR: erythrocyte sedimentation rate, ACPA: anti-citrullinated protein antibodies, RF: rheumatoid factor, VAS: visual analogue scale, DAS28: disease activity score-28

# Table S4 Multivariable predictors associated with a flare during DMARDs reduction

| Parameters/Markers | OR | 95% CI | P-value |
| --- | --- | --- | --- |
| Treg suppressive activity | 0.843 | 0.726–0.978 | 0.025* |
| DAS28 | 2.213 | 0.006–7.747 | 0.197 |
| VAS | 1.122 | 0.979–1.285 | 0.098 |

*Significance at p-value<0.05. VAS: visual analogue scale, DAS28: disease activity score-28

# Table S5 Clinical course of 9 patients regarding to symptom onset, disease duration, bone erosion and extra-articular manifestation

| Patient/Code | Symptom onset | Disease duration | Bone erosion | Extra-articular |
| --- | --- | --- | --- | --- |
| DT001 | 1 month | 6 years | no | no |
| DT002 | 1 year | 11 years | yes | no |
| DT003 | 3 months | 7 years | yes | no |
| DT004 | 2 months | 11 year | no | no |
| DT005 | 2 months | 5 years | yes | yes |
| DT006 | 1 month | 7 years | no | no |
| DT007 | 10 years | 11 years | yes | yes |
| DT008 | 6 months | 4 years | no | no |
| DT009 | 3 months | 7 years | no | no |


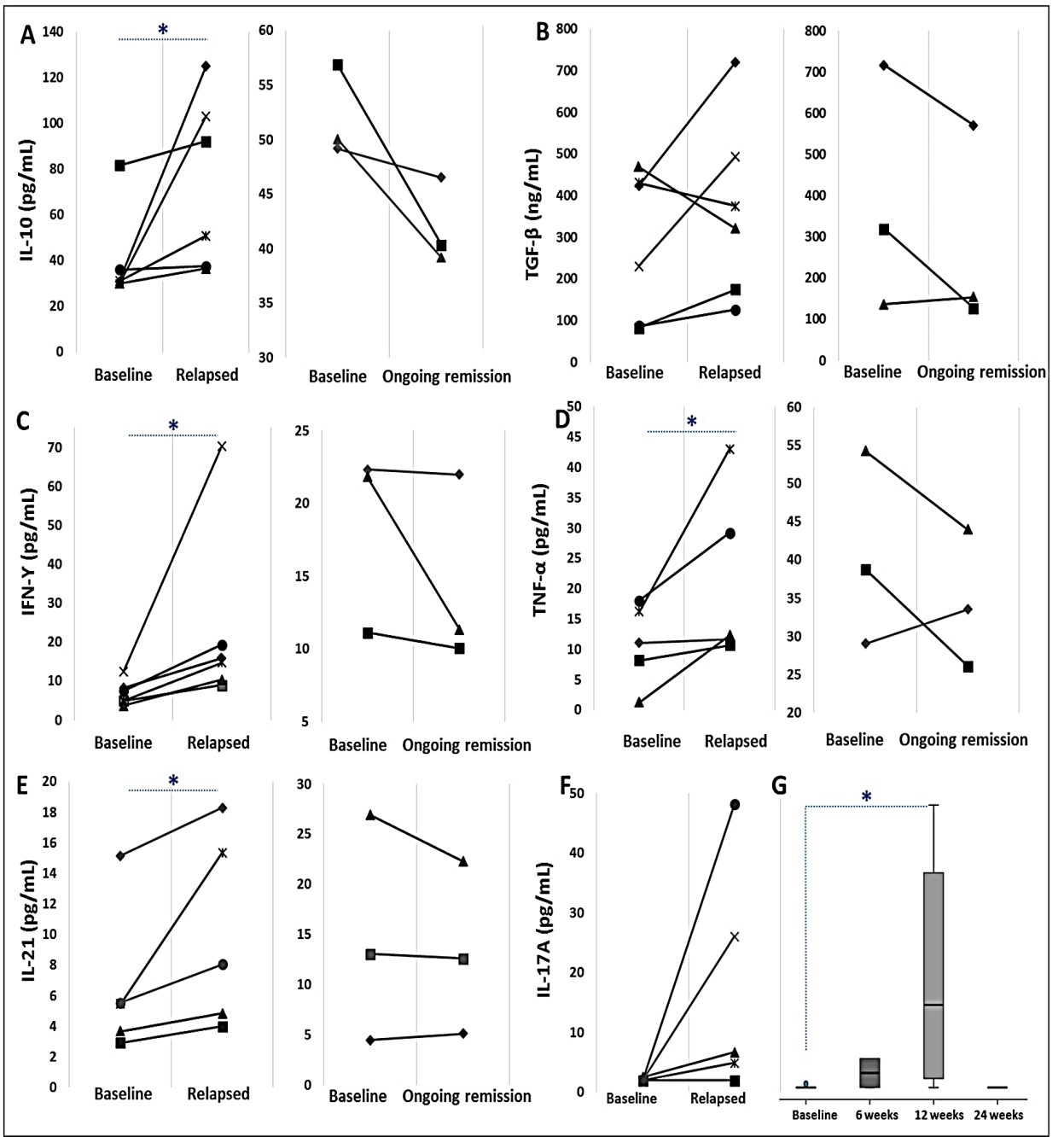


**Supplementary Figure 1.** Plasma cytokine levels in the peripheral blood of rheumatoid arthritis (RA) patients with disease-modifying anti-rheumatic drug (DMARD) dose reduction. (A) IL-10, (B) TGF-β, (C) IFN-γ, (D) TNF-α, (E) IL-21 and (F) IL-17A levels were determined at baseline and at relapse visits as well as during ongoing remission (at 24 weeks). These cytokine levels were compared between baseline and at disease relapse~~d~~ (left-hand side of each figure) and between baseline and ongoing remission (right-hand side of each figure). (G) IL-17A levels at different visits of patients with relapse at 6 and 12 weeks and of patients with ongoing remission at 24 weeks after DMARD dose reduction. Paired sample *t-*test analysis for TGF-β and Wilcoxon signed-rank test for other cytokines were conducted. Mann–Whitney U-test was used for IL-17A at different visits. **p* < 0.05.
